# The Liquid State of RIM1α and RBP Condensates is Maintained by Lipids

**DOI:** 10.1021/acsnano.5c07661

**Published:** 2025-11-21

**Authors:** Charlotte M. Fischer, Zenon Toprakcioglu, Ella de Csilléry, Gabriele S. Kaminski Schierle, Tuomas P. J. Knowles

**Affiliations:** † Centre for Misfolding Diseases, Yusuf Hamied Department of Chemistry, 2152 University of Cambridge, Lensfield Road, Cambridge CB2 1EW, United Kingdom; ‡ Department of Chemical Engineering and Biotechnology, 2152 University of Cambridge, Philippa Fawcett Drive, Cambridge CB3 0AS, United Kingdom

**Keywords:** biophysics, liquid−liquid phase separation (LLPS), liquid-to-solid transition, protein aggregation, lipid vesicles, amyloid formation

## Abstract

At the presynapse, RIM1α and RIM-binding protein (RBP) play a crucial role in regulating vesicle docking and priming. Emerging evidence suggests that these proteins are involved in the organization of the active zone where they cluster with membrane proteins and lipids, forming protein condensates driven by liquid–liquid phase separation (LLPS). While protein phase separation has been associated with cellular function, it has also been linked to various disorders, as liquid condensates can promote protein aggregation, leading to dysfunction. In this work, we investigated the phase behavior of RIM1α and RBP. We find that under physiological conditions and in the absence of crowding agents, RIM1α and RBP have the ability to spontaneously form biomolecular condensates. Moreover, these liquid condensates have the propensity to mature over time, resulting in a liquid-to-solid transition. Using a combination of fluorescence microscopy with biophysical techniques and characterization methods, we confirm that these solid aggregates are β-sheet-rich and fibrillar in nature. These observations not only add to the growing evidence that supports that RIM1α and RBP can phase separate, but we also show that these proteins can aggregate into fibrillar structures within condensates. Finally, we find that in the presence of lipid vesicles, this liquid-to-solid transition is suppressed, indicating the potential role that lipids play in maintaining the liquid state of RIM1α/RBP condensates. In the context of protein aggregation, these biophysical observations report on the mechanisms behind the phase separation and subsequent aggregation of RIM1α and RBP.

## Graphical abstract

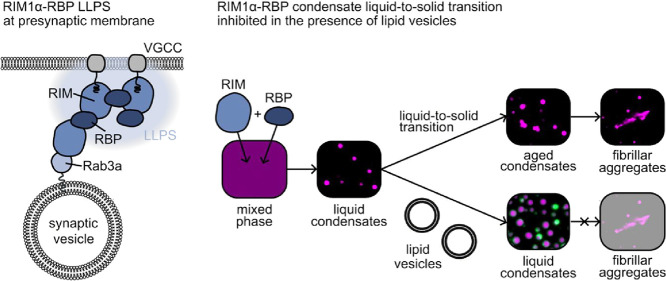

## Introduction

Synapses are essential components of the nervous system, serving as sites of information transmission, which is the foundation of learning, memory and cognitive processes. Dysregulation at the presynaptic level has been implicated in various neurodegenerative diseases, such as Alzheimer’s^1–4^ (AD) or Parkinson’s^5,6^ disease (PD). As such, studying presynaptic function can provide important insights into both neuronal function and the underlying causes of neurodegenerative disorders. Synaptic transmission involves the release of neurotransmitters from synaptic vesicles (SVs) into the synaptic cleft triggered by calcium influx into the presynapse as a response to an action potential.^7^ SV exocytosis is facilitated by the presynaptic active zone, a machinery of proteins and lipids aiding and regulating the docking of SVs to the presynaptic membrane and the release of neurotransmitters.^8,9^ To date, the molecular processes, such as SV docking, priming and fusion, performed by various proteins in the presynaptic active zone are not fully understood. While vesicular fusion is mediated by SNARE proteins,^10,11^ RIM1α as well as RIM-binding protein (RBP) and Rab3a have emerged as key proteins implicated in the regulation of SV trafficking.^12–16^

Liquid–liquid phase separation (LLPS) is a phenomenon in which proteins undergo demixing to form distinct, membraneless compartments within cells, driven by weak multivalent interactions.^17–19^ These dynamic condensates play crucial roles in cellular organization and processes,^20–25^ but their dysregulation has also been linked to disruption of cellular function,^26–30^ and more recently, to the formation of protein aggregates^31^ associated with neurodegenerative diseases, including PD^32^ and AD.^33–35^ It has been hypothesized that RIM1α/RBP liquid–liquid phase separation can drive SV localization to the presynaptic membrane by clustering voltage-gated calcium channels (VGCCs) with transmembrane proteins in SV membranes. RIM1α and RBP have also been shown to undergo liquid–liquid phase separation in vitro forming clusters with VGCCs on supported lipid membrane bilayers.^36,37^ In fact, increasing evidence suggests that lipids modulate phase separation for numerous different proteins, including proteins associated with neurodegenerative disorders.^38–41^

In this study, we investigate and characterize the RIM1α/RBP two-dimensional phase space, demonstrating that RIM1α and RBP can form biomolecular condensates in the presence of each other. Our findings provide evidence that under physiological concentrations, RIM1α and RBP condensates can undergo a liquid-to-solid transition and mature over time. This results in the development of solid-like structures within the condensates growing into elongated fibrillar aggregates. Moreover, we show that in the presence of small unilamellar lipid vesicles (SUVs), this liquid-to-solid transition is inhibited. These results indicate that the pairwise interaction of RIM1α and RBP in condensates, in addition to their function in SV exocytosis, could also play a role in disease-linked aberrant presynaptic aggregation processes.

## Results and Discussion

### RIM1α and RBP Undergo Phase Separation into Liquid-like and Solid-like Assemblies

Action-potential triggered neurotransmitter release requires the localization of synaptic vesicles next to voltage gated Ca^2+^ channels, which is mediated by the interactions of active zone proteins, including RIM1α and RBP. RIM1α and RBP can undergo liquid–liquid phase separation, a probable mechanism governing the organization of the presynaptic active zone.^36^ First, we examined the liquid–liquid phase separation propensity as a function of RIM1α and RBP2-(SH3)_3_ concentrations using fluorescence microscopy (Figures 1a and S1). RBP2-(SH3)_3_ is referred to as RBP throughout the rest of the manuscript, as it consists of the RIM-interacting SH3 domains of RBP with the fibronectin type III domains removed due to their unknown function. RIM1α and Cy3-RBP undergo phase separation at various concentrations ranging from 0.01 to 1 μM RIM1α and 0.1–1 μM RBP. Under the majority of conditions, liquid–liquid phase separation into round, spherical condensates was mostly observed. The domain structure of RIM1α and RBP has been previously studied^42–47^ and suggests that the liquid–liquid phase separation of RIM1α with RBP could be driven by the weak, but specific interactions of three proline-rich motifs (PRMs) of RIM1α with three SH3 domains located on RBP (Figure S2).^36,48^ This behavior is similar to other LLPS systems containing poly-PRM and poly-SH3 repeat peptides and proteins.^49–52^ Additionally, a sequence analysis of RIM1α and RBP using two machine-learning based disorder predictors, Metapredict^53^ and DeePhase,^54^ shows that both protein sequences are largely disordered, with an overall DeePhase score of 0.89 and 0.88 for RIM1α and RBP, respectively, where a score above 0.5 suggests that the protein is more likely than the average protein in the human proteome to undergo phase separation. Interestingly, at concentrations higher than 0.5 μM RIM1α and 0.5 μM RBP, we found mostly nonspherical protein aggregates alongside few condensates. To test how changes in the RIM concentration affect phase separation of the RIM1α/RBP system at concentrations below the detection limit of fluorescence microscopy (0.1 nM to 0.1 μM RIM1α), we performed diffusional sizing experiments (Figure S3). Diffusional sizing is a microfluidic technique, which allows the quantitative determination of the diffusion of a fluorescently labeled species by the acquisition of diffusion profiles at different time points. From this data, the diffusion constant and hydrodynamic radius of a species can be determined. Diffusional sizing can further be used to map the interaction of a solute with another solute species by measuring an increase in hydrodynamic radius upon binding. Here, we measured the binding of RIM1α to RBP at constant 0.01 μM Cy3-RBP, while varying the concentration of RIM1α between 0.1 nM and 0.1 μM. The gradual increase of the hydrodynamic radius with increasing RIM1α concentration indicated the formation of nanoclusters of RIM1α with RBP prior to the formation of liquid condensates seen at concentrations of more than 0.1 μM RIM1α. We further characterized the two-dimensional RIM1α/RBP phase space (Figure 1b). The phase diagram of the RIM1α/RBP system indicates three regimes: a mixed phase (where no phase separation was observed), a liquid–liquid phase separated phase regime, and an aggregate phase region. At higher RBP concentrations, the RIM1α concentration was shown to have a strong effect on the phase behavior. Conversely, at low concentrations of RIM1α no phase separation can be observed, while at higher concentrations, liquid condensates and eventually aggregates, were formed (Figure 1b). Protein aggregates, especially amyloid fibrils, have been associated with various neurodegenerative disorders. A thioflavin T (ThT) fluorescence-based assay can be used to detect fibrillar protein assemblies. Binding of ThT to fibrillar aggregates leads to an increase in the quantum yield of the fluorophore and subsequently ThT fluorescence can be used to monitor an increase in aggregate number.^55^ Here, we tested whether the observed protein aggregates could be distinguished from liquid condensates by ThT fluorescence (Figure 1c). Our results show that RIM1α/RBP aggregates were ThT active suggesting the presence of aggregates. Interestingly, at lower RIM1α and RBP concentrations of 0.5 μM RIM1α and 0.1 μM RBP liquid condensates were ThT inactive, while at high concentrations above 1 μM RIM1α or 1 μM RBP (Figure 1c) all protein assemblies were ThT active, indicating that the condensates had aggregated. These findings suggest that RIM1α and RBP could not only play a role in active zone organization, but also in aberrant protein aggregation processes taking place at the presynapse, leading to the disruption of presynaptic function or nucleation of amyloid fibrils.

**Figure 1:**
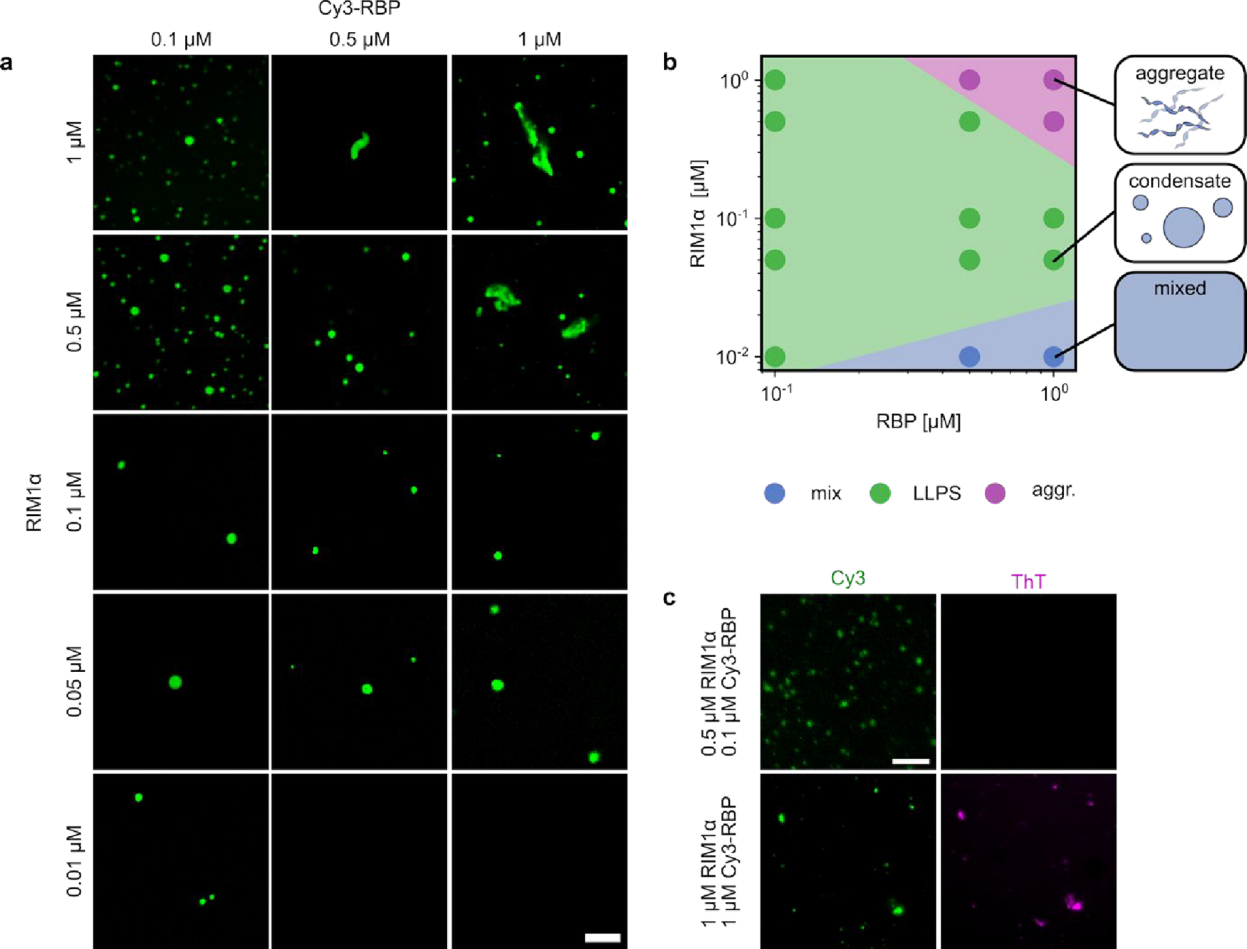
Characterization of the phase behavior of RIM1α and RBP as a function of the position in the phase space. (a) Phase separation of the RIM1α/RBP system. Fluorescence microscopic images of RIM1α/RBP condensates and aggregates at concentrations between 0.01 and 1 μM RIM1α and 0.1–1 μM Cy3-RBP. The scale bar is 5 μm. (b) Phase diagram of RIM1α/RBP showing mixed, liquid–liquid phase-separated, and aggregated regions of the two-dimensional phase space. (c) Fluorescence imaging of Cy3-RBP and ThT fluorescence of RIM1α/RBP protein assemblies at 0.5 μM RIM1α, 0.1 μM Cy3-RBP and 1 μM RIM1α, 1 μM Cy3-RBP, respectively. Images were acquired immediately after mixing RIM1α and RBP. The scale bar is 30 μm.

### RIM1α/RBP Condensates Undergo a Liquid-to-Solid Transition into Fibrillar Aggregates over Time

Recent studies show that intrinsically disordered, phase separating proteins, such as Tau,^56^ α-synuclein,^57,58^ amyloid-β,^59^ FUS,^60^ TDP-43 ^61^ or hnRNPA1 ^62^ can undergo gelation or aggregation forming solid-like fibrils via liquid–liquid phase separation. These results suggest that a functional liquid–liquid phase separated state could facilitate a pathological liquid-to-solid transition into protein aggregates. Here, we investigated whether the formation of RIM1α/RBP aggregates is facilitated by the prior formation of RIM1α/RBP liquid condensates. To test this, we performed fluorescence recovery after photobleaching (FRAP) experiments on fresh and aged RIM1α/RBP condensates, to assess the dynamics in the dense phase (Figure 2a–c). Interestingly, we find that following photobleaching, the Cy3-RBP fluorescence only recovers up to 25% for fresh condensates after 550 s, suggesting a low mobility of RBP in the dense phase at early time points. Further, aged condensates do not recover (<5%) from photobleaching, indicating solid-like material properties. Additionally, to investigate the reversibility of fresh and aged condensates, we performed a dissolution assay of RIM1α/RBP condensates (Figures 2d and S4) in order to corroborate our previous findings and further probe which types of interactions play a role in RIM1α/RBP phase separation. In a dissolution assay, liquid–liquid phase separation disruptors are used to determine the reversibility of condensate formation – reversibility suggesting liquid-like and irreversibility suggesting more solid-like behavior – and the nature of the solute interactions that lead to phase separation. Liquid condensates formed mainly by hydrophobic interactions are dissolved by hydrophobic disruptors such as 1,6-hexanediol,^63^ and liquid condensates formed through electrostatic interactions are dissolved by charge screening, for example by using high salt solutions.^64^ However, aged condensates are in a metastable state and either cannot be dissolved, or dissolve only on significantly larger time scales. Here, we tested whether RIM1α/RBP condensates formed immediately and after 8d can be reversed by adding phase separation disruptors, exhibiting either liquid or solid-like properties. Fresh condensates were completely dissolved using 1 M 1,6-hexanediol and partially dissolved using 0.3 M KCl, indicating that the condensates are mostly stabilised by hydrophobic protein interactions, likely interactions of the proline-rich motives of RIM1α with the hydrophobic binding pockets of the SH3 domains of RBP,^65–67^ and partially supported by electrostatic interactions. Dissolution or partial dissolution, was observed immediately after addition of the dissolution agent to the phase separated sample, showcasing the liquid-like behavior of the condensates. In comparison, matured condensates could not be dissolved under the same conditions, indicating that the initially liquid-like condensates have adapted more solid-like properties. Our conclusions demonstrate both that a liquid-to-solid transition of RIM1α/RBP condensates takes place over time and that the predominant force holding the condensates together are hydrophobic interactions. In comparison, other phase separating proteins such as FUS and TDP-43, are held together by hydrophobic and electrostatic interactions equally, as they can be dissolved by hydrophobic or electrostatic disruptors.^64^

**Figure 2:**
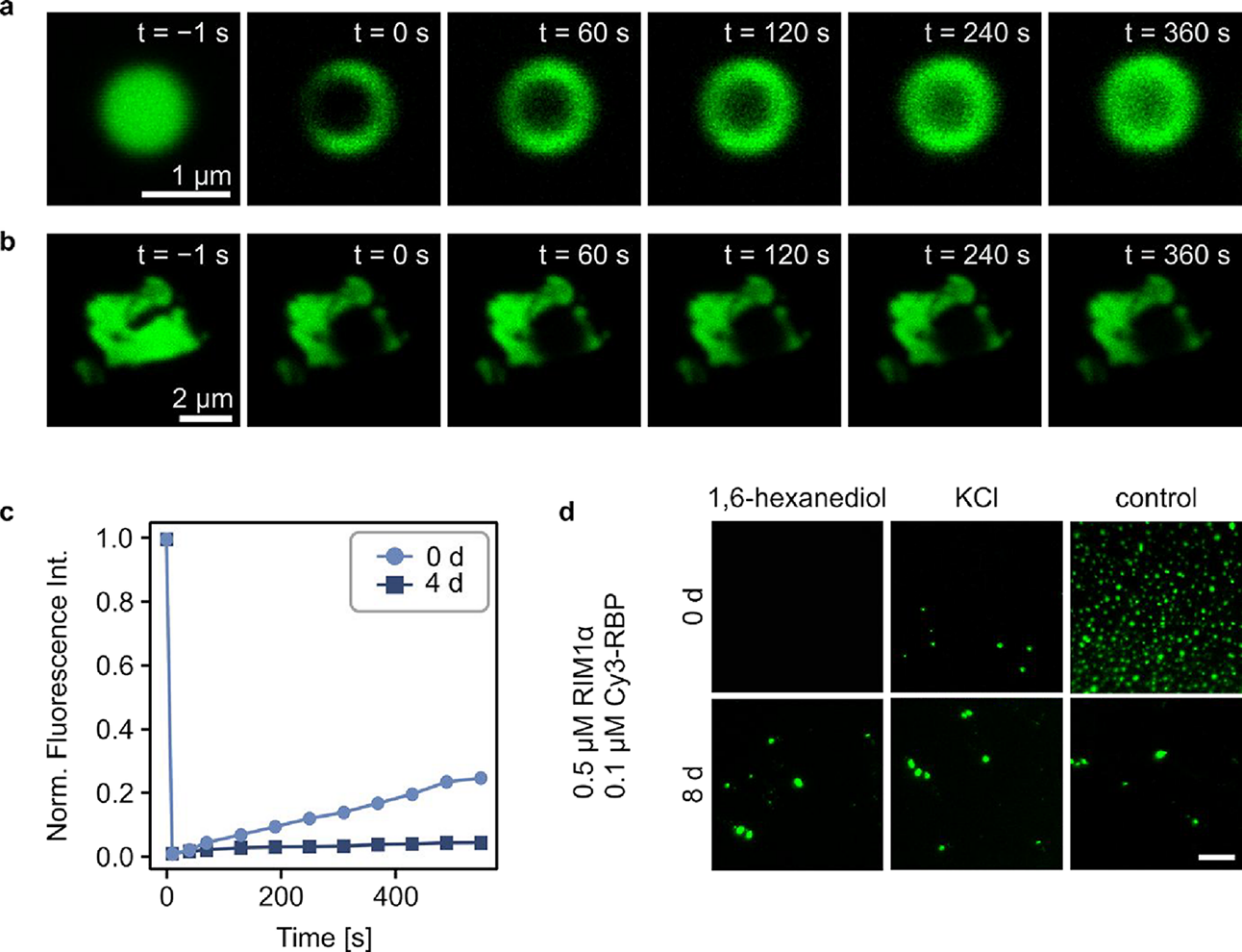
RIM1α/RBP condensates undergo a liquid-to-solid transition over time. (a) Fluorescence recovery after the photobleaching experiment performed on fresh RIM1α/RBP condensates. (b) Fluorescence recovery after the photobleaching experiment performed on aged RIM1α/RBP condensates. (c) Fluorescence recovery curve of fresh and aged RIM1α/RBP condensates. (d) Fluorescence imaging of dissolution of new and matured RIM1α/RBP condensates. Dissolution of RIM1α/RBP condensates with 1,6-hexanediol or KCl immediately after sample preparation and after 8 d. The scale bar is 40 μm.

Furthermore, we investigated the morphology of the RIM1α/RBP condensates as they mature and age over time. RIM1α/RBP liquid condensates were placed in a sealed capillary and imaged over a 20 h period (Figure 3a). Initially, from the liquid–liquid phase separated solution, nucleation was observed after 5 h, followed by growth of fibrils. This was monitored again through the use of ThT fluorescence. An increase in intensity was observed indicating a transition from random coil to β-sheet structures. This transition was confirmed by Fourier transform infrared spectroscopic (FTIR) measurements. Spectra were acquired of RIM1α/RBP condensate solutions at *t* = 0 h and after Incubation for 24 h. These are shown in Figure S5. As can be seen in the spectra, there is a shift of both the amide I and II bands toward lower wavenumbers over time. Specifically, the amide I band shifts from 1655 to 1635 cm^–1^, while the amide II band shifts from 1550 to 1540 cm^–1^. These are indicative transitions of random coil to β-sheet. Additionally, transmission electron microscopy (TEM) was used to investigate the morphology and structure^68^ of the RIM1α/RBP aggregates following 23 d of incubation (Figure 3b), which determined that RIM1α/RBP aggregates grow to sizes of about 1 μm in length and are comprised of a fibrillar morphology. Like the majority of aggregate structures from intrinsically disordered proteins, including amyloid-β, α-synuclein or FUS, the width of the identified fibrils was in the order of nanometres. Our findings suggest that RIM1α/RBP aggregate formation shares structural similarities with these aggregates, and the transition from a liquid to a solid state may contribute to protein dysfunction.

**Figure 3:**
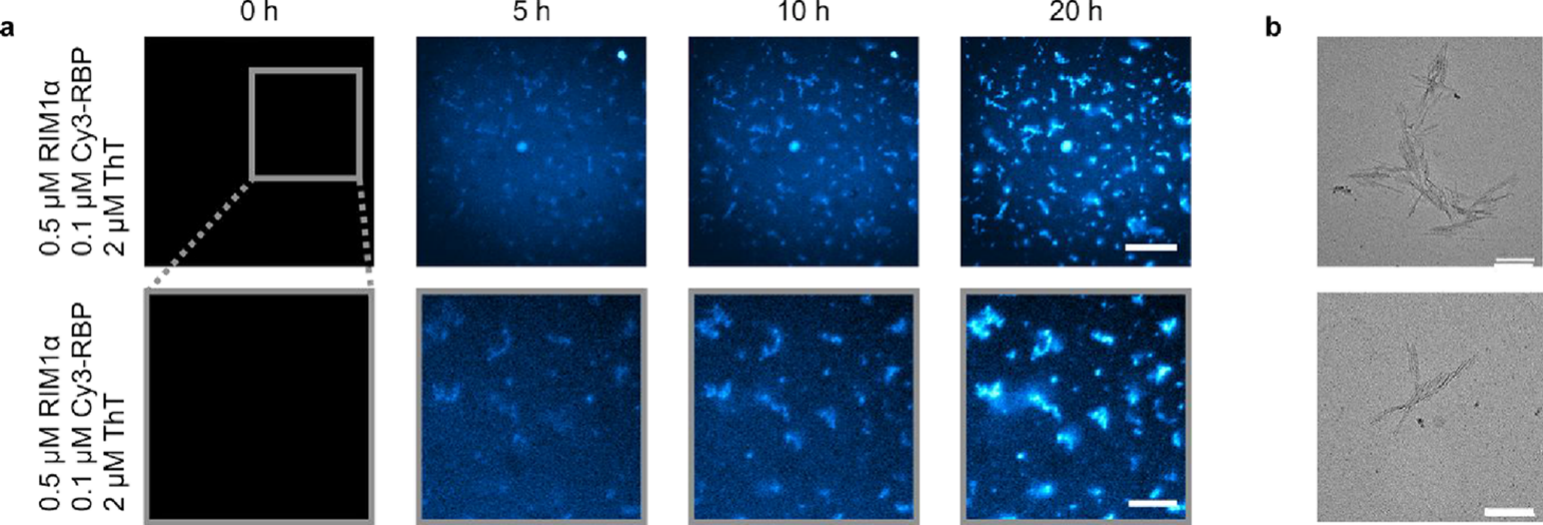
RIM1α and RBP can form fibrillar aggregates over time. (a) ThT fluorescence images of RIM1α/RBP aggregate nucleation and growth from the liquid–liquid phase separated phase at 0, 5, 10, and 20 h. Scale bars are 80 μm (top row) and 30 μm (bottom row). (b) TEM imaging of 23 d old fibrillar RIM1α/RBP aggregates from a sample of 0.5 μM RIM1α, 0.1 μM RBP. Aggregates were grown at room temperature without shaking. The scale bar is 200 nm.

### RIM1α/RBP Liquid-to-Solid Transition over Time Is Inhibited by SUVs

RIM1α and RBP are involved in synaptic vesicle trafficking and exocytosis at the presynaptic membrane, suggesting a direct interaction with synaptic vesicles.^12–16^ Previous work has shown that lipids such as those present in synaptic vesicles, can strongly influence protein phase behavior and function.^38–41^ To explore the crucial role that lipids have on controlling protein function, we investigated the effect of small unilamellar lipid vesicles (SUVs) on the phase separation and liquid-to-solid transition of RIM1α and RBP. SUVs resemble the size, curvature and dynamics of synaptic vesicles and as such, present a good model system to study the interaction of biomolecular condensates with lipids in vitro*.* A lipid composition of DOPC:DOPE:DOPS:Cholesterol of 18:45:27:10 was chosen to reflect the native composition of synaptic vesicles.^69^ A mixture of RIM1α/Cy3-RBP droplets was prepared with 100 μM SUVs. The solution was placed in a sealed capillary and imaged over time for 24 h (Figure 4a). We found that in the presence of lipid SUVs, RIM1α and RBP condensates remain spherical, are mobile in solution and do not form fibrillar aggregates, suggesting liquid-like material properties and indicating that SUVs play a role in inhibiting the liquid-to-solid transition of RIM1α/RBP condensates. Further, we tested whether the condensates formed in the presence of SUVs can be dissolved by addition of 1,6-hexanediol (Figure 4b) after 0 and 24 h of incubation. Interestingly, we found that the condensates do not dissolve upon the addition of 1,6-hexanediol, indicating that the lipids might shield the proteins from interacting with small molecules such as 1,6-hexanediol. In comparison, SUVs formed without cholesterol (i.e., DOPC:DOPE:DOPS 18:50:30) similarly preserve the liquid state of RIM1α/RBP condensates as we still observed spherical condensates over time (Figure S6). However, when 1,6-hexanediol was added to the condensate-SUV solution, dissolution of the condensates was observed (Figure S6). This suggests that cholesterol, a main component in synaptic vesicles, is responsible for preventing interactions on the condensate surface. Further work is needed to fully unravel the mechanism of this phenomenon. A potential mode of action which would account for our experimental results is that cholesterol increases the affinity of RIM1α and RBP condensates to interact with the surface of the lipid bilayer. If this is indeed the case, this enhanced affinity could effectively reduce the interaction of small molecules such as 1,6-hexanediol with the condensates, explaining why no condensate dissolution with SUVs containing cholesterol was observed. Alternatively, the 1,6-hexanediol itself could interact and partition into the SUVs, thereby resulting in a decrease of the effective 1,6-hexanediol concentration in bulk. This in turn could reduce the effect of 1,6-hexanediol on the RIM1α/RBP condensates which could also explain why the condensates are not dissolved. It is therefore evident that the lipid membrane properties play a fundamental role in modulating condensate interactions. Even small changes in the composition of the SUVs, such as the addition of cholesterol in this particular case, can have significant changes in the manner by which proteins behave and interact with vesicles. More research into this exciting new area is clearly needed if we are to fully understand LLPS at the molecular level.

**Figure 4:**
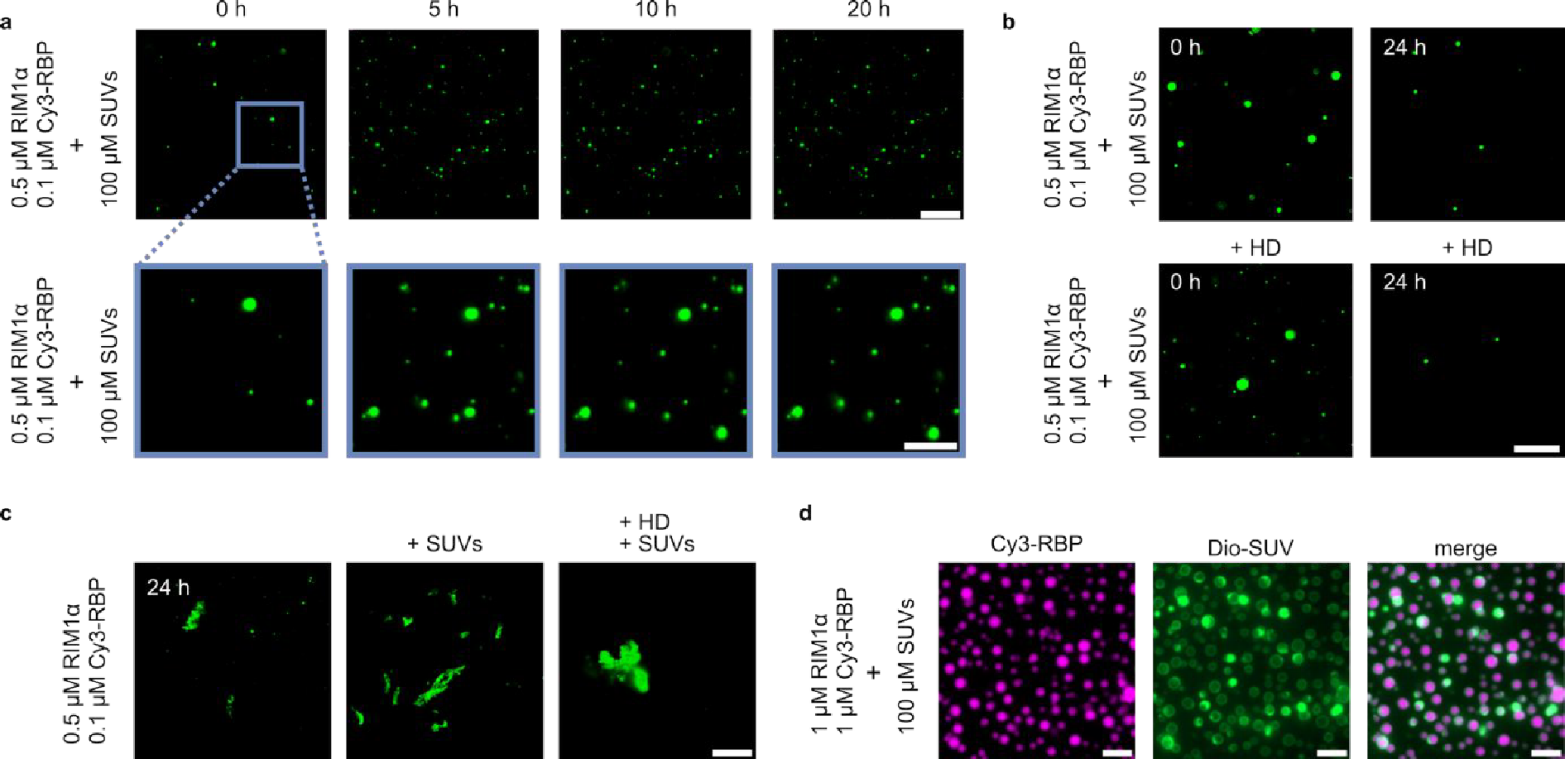
Inhibition of RIM1α/RBP liquid-to-solid transition by SUVs. (a) Cy3-fluorescence images of the RIM1α/Cy3-RBP liquid–liquid separated phase in the presence of 100 μM SUVs at 0, 5, 10, and 20 h. Scale bars are 50 μm (top row) and 20 μm (bottom row). (b) Fluorescence images of the RIM1α/Cy3-RBP sample incubated with 100 μM SUVs for 0 and 24 h before and after addition of 1,6-hexanediol. The scale bar is 20 μm. (c) Fluorescence images of 24 h old RIM1α/RBP condensates incubated without SUVs (left panel), after adding 100 μM SUVs (middle panel), and after adding 100 μM SUVs with 1,6-hexanediol. For all experiments, the SUVs and/or 1,6-hexanediol was added following 24 h incubation of the condensates. The scale bar is 20 μm. (d) Cy3-RBP and Dio-SUV fluorescence images of fresh RIM1α/RBP condensates in the presence of 100 μM SUV. The scale bar is 5 μm.

We further explored the possibility of SUVs to revert the solid condensates back to their liquid state. To do this, SUVs were added to aged RIM1α/RBP condensates. We found that the condensates remain insoluble (Figure 4c), indicating that SUVs are not able to reverse the liquid-to-solid transition. This suggests that SUVs interact and stabilize the liquid protein condensates, inhibiting them from transitioning to an aggregated state. The fact that lipids had no influence on the solid, aged condensates suggests that this interaction occurs at the monomeric protein level. To investigate how lipid vesicles could potentially suppress the liquid-to-solid transition, we studied the localization of the lipids interacting with RIM1α/RBP condensates (Figure 4d) by using fluorescently labeled Dio-SUVs. It has been reported that nucleation of fibrils often occurs first at the interface^54^ of condensates as previously shown for protein systems such as FUS^70^ and tau^56^ and lipids could play a role in interfering with these nucleation events. Interestingly, we find that the lipid vesicles coat on the surface of RIM1α/RBP condensates, suggesting that they potentially contribute to suppressing the nucleation of fibrils at the condensate interface.

## Conclusions

In this work, we explore the phase separation of RIM1α with RBP under physiological conditions and identify liquid and solid-like phase behaviors in distinct regions across the phase space. We further characterize these biomolecular condensates formed by liquid–liquid phase separation of RIM1α with RBP and show that they have the ability to undergo a liquid-to-solid transition at physiological concentrations over time. Using FRAP and condensate dissolution assays involving 1,6-hexanediol, we demonstrate that the dynamics and reversibility of liquid condensates are reduced as condensates mature. Furthermore, using FTIR spectroscopy, we show that these aggregates undergo a random coil to β-sheet transition and, using electron microscopy, we confirm that indeed they exhibit a fibrillar morphology. Moreover, we monitored the nucleation and growth of these aggregates over time using ThT fluorescence. Such processes could have implications in a variety of diseases and conditions, including neurodegenerative disorders, where protein aggregates and fibrils are observed.

We further explored how lipid vesicles could influence the phase separation of RIM1α with RBP. Interestingly, we find that the presence of lipid vesicles (SUVs) – with a composition resembling the lipid composition of synaptic vesicles – inhibits the liquid-to-solid transition of RIM1α and RBP. We find that lipid vesicles coat the RIM1α/RBP condensates, suggesting a potential regulatory role in suppressing the nucleation of RIM1α/RBP fibrils at the condensate interface and maintaining a functional liquid-like state. Indeed, aggregation commonly initiates at the interface of condensates,^70–72^ and as such passivation of this interface is likely to be a key mechanism to suppress aggregation within biomolecular condensates. Interestingly, an inhibitory role of lipids in the aggregation of amyloidogenic proteins has been reported in other contexts.^73^ Taken together, these results highlight the biophysical principles by which lipids can maintain the liquid nature of biomolecular condensates and prevent aberrant aggregation.

## Materials and Methods

### Protein Expression

RIM1α and sortase Δ59 were expressed and purified (Figure S7) using a previously established protocol from Wu et al.^36^ The plasmids were kindly gifted from Prof. Mingjie Zhang at Hong Kong University of Science and Technology. In brief, different fragments of RIM1α were amplified using standard PCR and cloned into modified pET-32a vectors containing an N-terminal Trx-His_6_ tag and either an HRV-3C or TEV protease cleavage site. The two fragments RIM1α-N(1–474) with a C-terminal LPETGG-tag and RIM1α-N(483–1334) as well as sortase Δ59 (including a Trx-His6 tag) were expressed in *E. coli* BL21­(DE3) cells (Agilent Technologies). Both RIM1α constructs include a N-terminal Trx-His6 tag and an HRV-3C protease cutting site (fragment RIM1α-N(1–474)) or a TEV protease cutting site (RIM1α-N(483–1334)). The cells were cultured in LB medium at 16 °C. Purification was performed using a Ni^2+^-NTA affinity column, followed by size exclusion chromatography on a Superdex 200 16/60 column. The affinity tag was removed by overnight digestion at 4 °C with either HRV-3C or TEV protease. A second round of Superdex 200 16/60 size exclusion chromatography was carried out to eliminate the cleaved tag, using a buffer composed of 50 mM Tris (pH 8.2), 200 mM NaCl, 1 mM EDTA, and 1 mM DTT. Purified RIM1α-N(1–474) was combined with RIM1α-N(1–474) and sortase Δ59 in a 2:1:1 molar ratio. The ligation reaction was initiated by adding 10 mM CaCl_2_ and incubating at room temperature for 2 h. The resulting RIM1α-FL was separated from unreacted components using Superdex 200 26/60 size exclusion chromatography in a buffer containing 50 mM Tris (pH 8.2), 300 mM NaCl, 1 mM EDTA, and 1 mM DTT.

RBP2-(SH3)_3_ (referred to as RBP) was expressed and purified following a previously established protocol by Wu et al.^36^ (Figure S8). This protein was generated by fusing the first SH3 domain of rat RIM-BP2 (GenBank: XM_017598284.1) with its C-terminal SH3 tandem. It was tagged with His_6_, separated by an HRV3C protease cleavage site (designated His_6_-HRV-RBP2-(SH3)_3_). The gene encoding the target protein was cloned into the pET29b vector for recombinant expression in *E. coli* BL21­(DE3) (Agilent Technologies) following transformation. The plasmid was kindly provided by Prof. Mingjie Zhang from the Hong Kong University of Science and Technology. Protein expression was induced with IPTG and carried out using standard methods. Purification was performed via nickel affinity chromatography (Cytiva HisTrap Excel, 5 mL), followed by size exclusion chromatography (SEC) on a Superdex 200 16/60 column in a buffer containing 50 mM HEPES (pH 7.5), 300 mM NaCl, and 1 mM TCEP. To cleave the His_6_ tag, the His_6_-HRV-RBP2-(SH3)_3_ protein was concentrated, 3 mol % His_6_-HRV protease (50 μM, 30 μL) was added and the mixture was incubated overnight at 4 °C. Finally, a reverse nickel chromatography was carried out.

### Fluorescence Imaging

A Leica Stellaris 5 confocal microscope equipped with a 63× oil immersion Leica 1.4 NA objective or an inverted Zeiss microscope with a 60x oil immersion objective were used for imaging with an excitation filter at 546 nm (Cy3) and 488 nm (ThT, Dio), respectively. Unless indicated differently, the samples were imaged by depositing an appropriate amount of sample on a glass slide and imaging immediately.

### LLPS and Aging Assays

To induce phase separation of RIM1α and RBP the purified proteins were added to an appropriate amount of buffer from the protein stock solutions and mixed by pipetting. In all experiments, the buffer contained 20 mM HEPES pH7.5, 100 mM NaCl and 3 mM TCEP. The samples were prepared and incubated for 5 min in sealed tubes prior to imaging. Aged samples were prepared in a similar way and incubated for longer periods of time as indicated. For experiments using ThT, samples were prepared in a similar way and 50 μM ThT were added to the sample before mixing. In experiments with lipid SUVs, 100 μM lipid-SUVs were added to the sample before mixing. Dissolution experiments were carried out by preparing the sample as described above, then adding 1.6 M 1,6-hexanediol or 0.3 M KCl, respectively, mixing by pipetting and incubating for 5 min, or longer if indicated, prior to imaging. Experiments monitoring the liquid-to-solid transition of RIM1α and RBP over time were carried out in sealed glass capillaries.

### Fluorescence Recovery After Photobleaching (FRAP)

Fluorescence recovery after photobleaching (FRAP) experiments were conducted using a Leica Stellaris 5 confocal microscope equipped with a 63× oil immersion objective (Leica HC PL APO 63×/1.40 Oil CS2, NA 1.4). All images were acquired at a resolution of 1024 × 1024 pixels. A 561 nm argon laser was used to photobleach a circular region within the condensates, with bleaching performed at full laser power for 10 s. Postbleach imaging included 2 frames taken at 30-s intervals, followed by 15 frames at 60-s intervals. Data were processed using Fiji software (version 2.16.0), with double normalization applied using background and reference regions of interest (ROIs).

### Fourier Transform Infrared Spectroscopy (FTIR)

To confirm β-sheet formation and monitor conformational changes in RIM1α and RBP, we employed Fourier-transform infrared (FTIR) spectroscopy. All measurements were carried out using an Equinox 55 spectrometer (Bruker). Samples were placed on the FTIR sample holder, and spectra were corrected by subtracting an appropriate buffer reference along with compensation for atmospheric carbon dioxide. Each final spectrum was obtained by averaging 256 scans. All FTIR experiments were conducted under ambient conditions.

### Transmission Electron Microscopy

Samples were imaged using transmission electron microscopy (TEM). The mixture of RIM1α and RBP was left to age for a week, before being imaged. 5 μL of sample was spotted onto carbon films consisting of 3 mm 300-mesh copper grids (EM Resolutions Ltd.). This sample was subsequently stained with 5 μL of 2% (w/v) uranyl acetate. Finally, TEM micrographs were acquired using a FEI Talos F200X G2 transmission electron microscope.

### Preparation of Liposomes

A 100 mM lipid solution was prepared. DOPC, DOPE, DOPS and cholesterol (Avanti Polar Lipids), were initially dissolved in chloroform and then mixed in a molar ratio of 18:45:27:10. Another solution was prepared in the absence of cholesterol, with a composition of DOPC, DOPE and DOPS (18:50:30), respectively. The remainder of the solution consisted of the lipophilic tracer dye Dio (Avanti Polar Lipids), which was used to label the liposomes. The chloroform was then evaporated, resulting in the formation of a film. This was subsequently rehydrated with the appropriate buffer before being subjected to repeat freeze–thaw cycles. The liposomes were finally prepared through sonication (Bandelin Sonopuls HD 2070 homogenizer) for 15 min. To ensure uniform liposome size, the solution was repeatedly extruded using a 30 nm pore size membrane. The liposomes were then mixed with proteins before being imaged.

## Supplementary Material



## Data Availability

The raw data and analysis code underlying this study will be made available upon request.

## Notes

Additional images of the phase separation of the RIM1α/RBP system; sequence-based analyses of RIM1α and RBP domain structures and intrinsic disorder scores; microfluidic diffusional sizing analysis of the RIM1α/RBP system; additional fluorescence images of the dissolution of RIM1α/RBP condensates; FTIR spectra of RIM1α/RBP condensate solutions; inhibition of RIM1α/RBP liquid-to-solid transition by SUVs (DOPC:DOPE:DOPS 18:50:30); preparation of RIM1α -FL; and preparation of RBP (PDF)
