# Supplementary material for: The Liquid State of RIM1α and RBP Condensates is Maintained by Lipids
Source: ACS Nano. 2025 Nov 21;19(48):40789–97. doi: 10.1021/acsnano.5c07661 (PMC12874637; doi:10.1021/acsnano.5c07661)
Supplement: Supplementary file 1 [file nn5c07661_si_001.pdf]

Supporting Information:
The liquid state of RIM1α and RBP condensates is maintained by lipids

Charlotte M. Fischer,[1, †] Zenon Toprakcioglu,[1, †] Ella de Csilléry,[1, †] Gabriele S. Kaminski Schierle,[2, *] Tuomas P. J. Knowles [1, *]

[1] Centre for Misfolding Diseases, Yusuf Hamied Department of Chemistry, University of Cambridge, Lensfield Road, Cambridge CB2 1EW, United Kingdom
[2] Department of Chemical Engineering and Biotechnology, University of Cambridge, Philippa Fawcett Drive, Cambridge CB3 0AS, United Kingdom

[†] Contributed equally
[*] Correspondence should be addressed to gsk20@cam.ac.uk or tpjk2@cam.ac.uk

Supplementary Figures

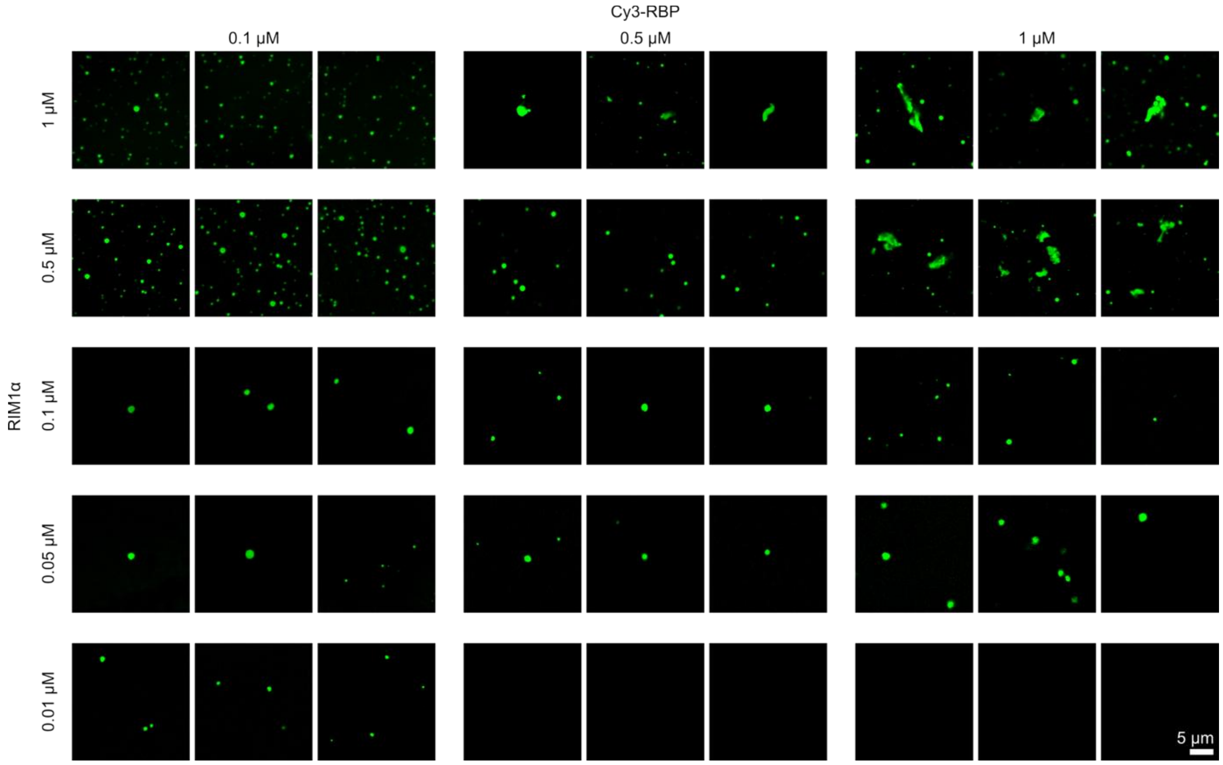

Figure S1. Additional images of the phase separation of the RIM1α/RBP system. Fluorescence images of RIM1α/RBP condensates and aggregates were obtained at concentrations between 0.01-1 µM RIM1α and 0.1-1 µM Cy3-RBP. Scale bar is 5 µm.

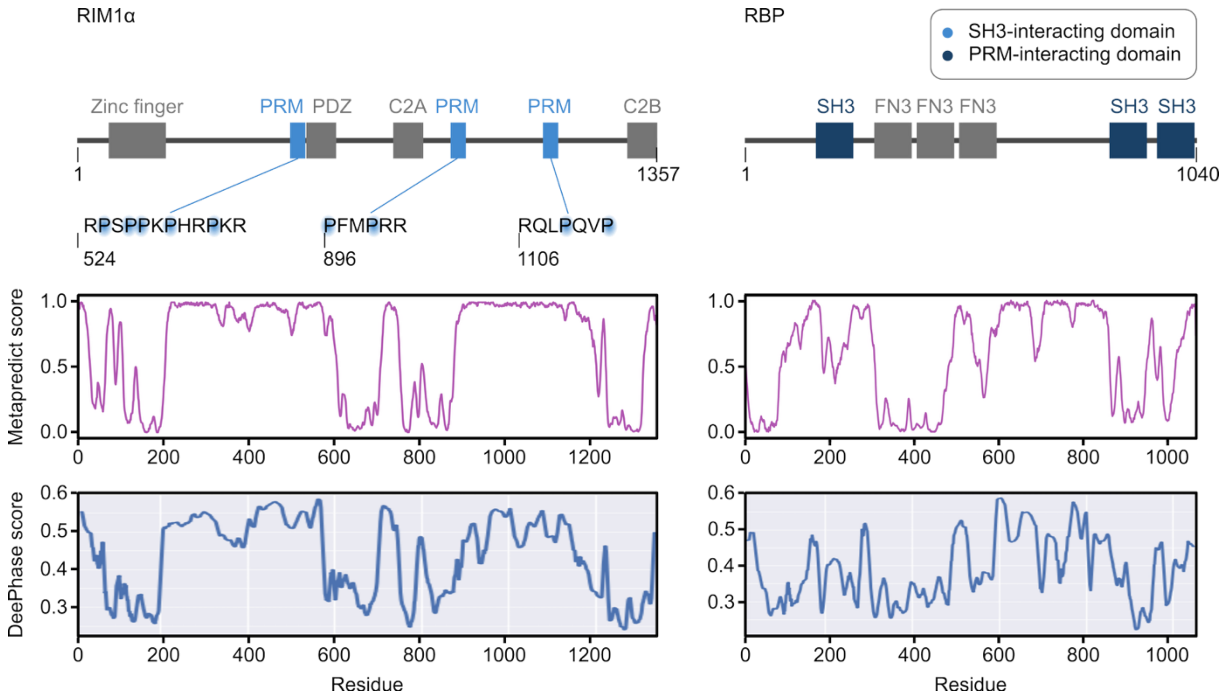

Figure S2. Sequence analysis of RIM1α and RBP. (a) Domain structure of RIM1α and RBP. Domains responsible for specific interactions of the two proteins, proline-rich motifs on RIM1α and SH3 domains on RBP, respectively, are highlighted in blue. (b) Metapredict and DeePhase disorder scores of RIM1α and RBP as a function of the sequence residue.

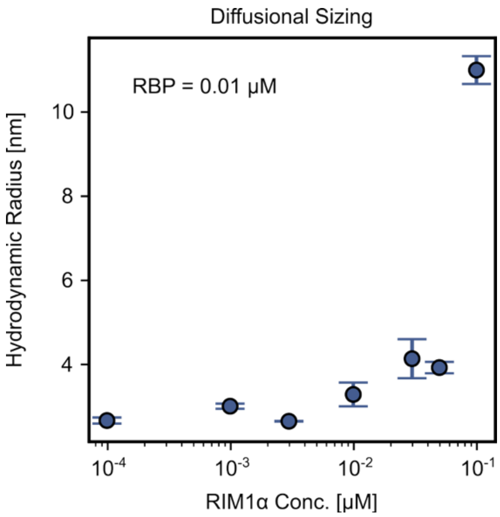

Figure S3. Microfluidic diffusional sizing analysis of RIM1α and Cy3-RBP at a fixed Cy3-RBP concentration. The observed increase in hydrodynamic radius suggests a possible interaction between RIM1α and RBP followed by subsequent nanocluster formation and phase separation occurring at RIM concentrations exceeding 0.05 µM.

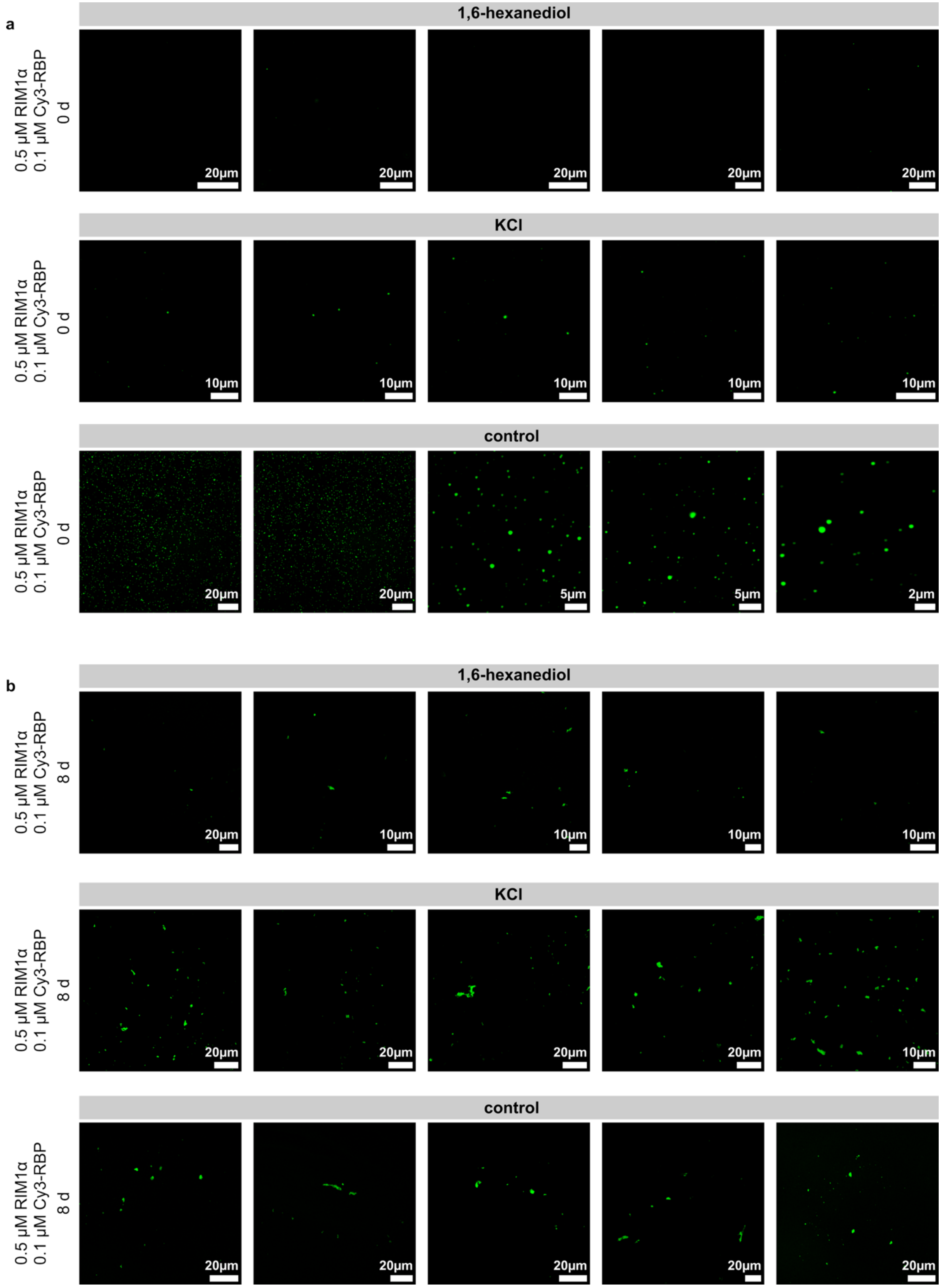

Figure S4. Additional fluorescence images of the dissolution of new and matured RIM1α/RBP condensates. (a) Dissolution of RIM1α/RBP condensates with 1,6-hexanediol or KCl immediately after sample preparation (b) Dissolution of RIM1α/RBP condensates with 1,6-hexanediol or KCl after 8d.

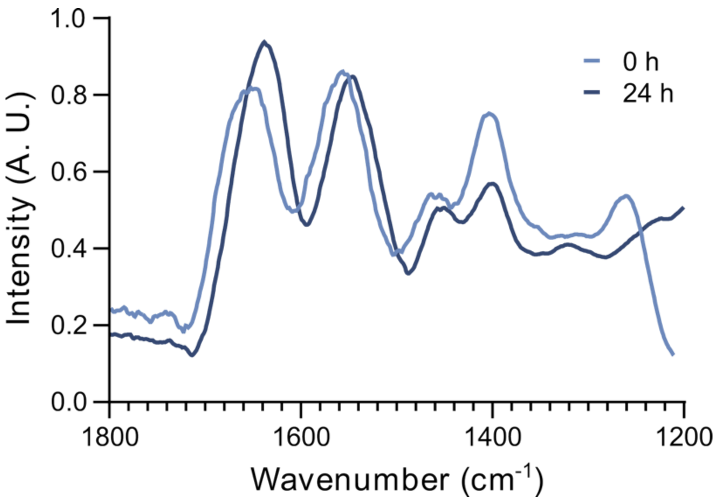

Figure S5. FTIR spectra acquired of RIM1α/RBP condensate solutions after 0 and 24 h of incubation.

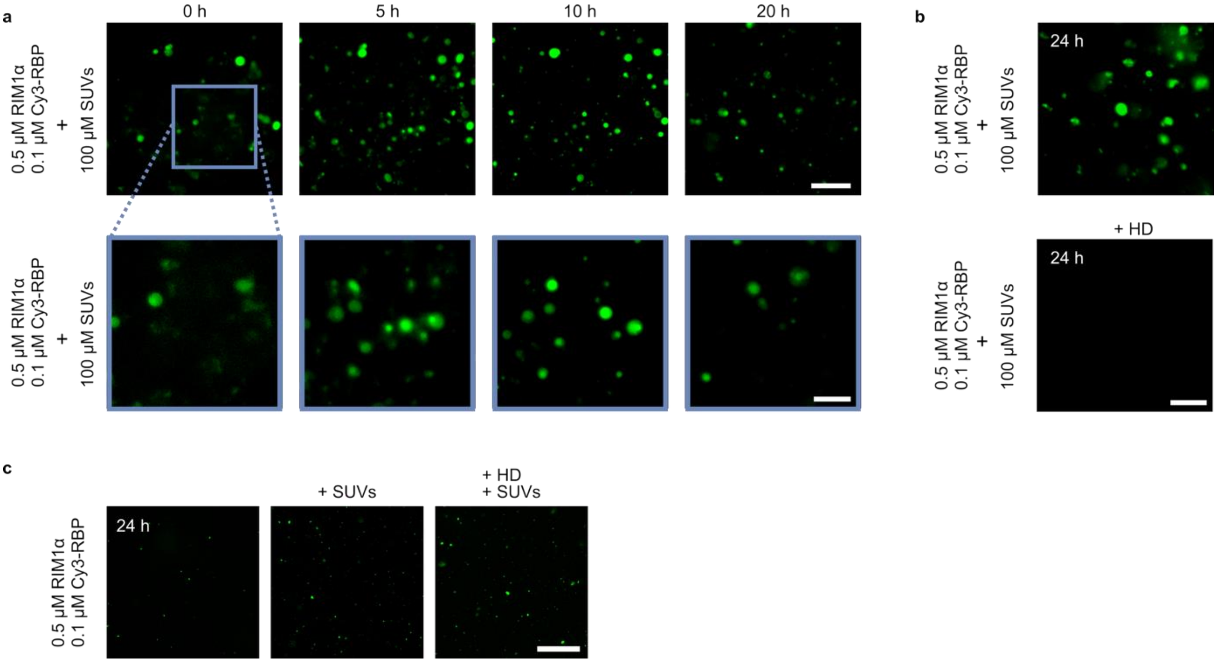

Figure S6. Inhibition of RIM1α/RBP liquid-to-solid transition by SUVs (DOPC:DOPE:DOPS 18:50:30). (a) Cy3-fluorescence images of RIM1α/Cy3-RBP liquid-liquid phase separated phase in the presence of 100 µM SUVs at 0, 5, 10 and 20 h, respectively. Scale bars are 50 µm (top row) and 20 µm (bottom row). (b) Fluorescence images of RIM1α/Cy3-RBP sample incubated with 100 µM SUVs for 24 h before and after addition of 1,6-hexanediol. Scale bar is 20 µm (c) Fluorescence images of 24 h old RIM1α/RBP condensates incubated without SUVs (left panel), after adding 100 µM SUVs (middle panel), and after adding 100 µM SUVs with 1,6-hexanediol. For all experiments, the SUVs and/or 1,6-hexanediol was added following 24 h incubation of the condensates. Scale bar is 20 µm.

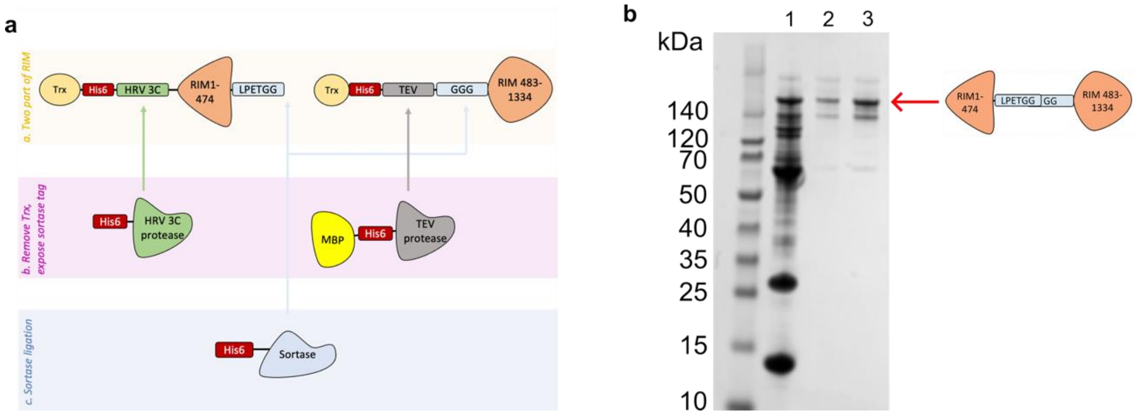

Figure S7. Preparation of RIM1α-FL using the sortase ligation method protocol described in [1]. (a) Schematic showing the three-step process: RIM1α was expressed in two fragments, RIM1α -N(1-474) and RIM1α -N(483-1334). In the second step, the linkers were cleaved using Trx and TEV protease to expose the sortase binding sites. In the final step, the two fragments were combined by sortase ligation. (b) SDS-PAGE analysis of the crude RIM-mixture after sortase ligation (1) and after size exclusion chromatography (SD200 16/60) (2,3).

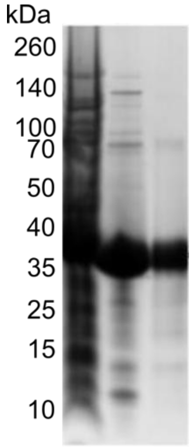

Figure S8. Preparation of RBP using the protocol described in [1]. SDS-PAGE analysis of the crude RIM-mixture after cell lysis (1), after Nickel elution (2) and after size exclusion chromatography (SD200 16/60) (3).

References

1. Wu, X. *et al.* RIM and RIM-BP Form Presynaptic Active-Zone-like Condensates via Phase Separation. *Mol Cell* 73, 971-984.e5 (2019).
